# Modeling and optimization of dyeing process of polyamide 6 and woolen fabrics with plum-tree leaves using artificial intelligence

**DOI:** 10.1038/s41598-024-64761-7

**Published:** 2024-07-02

**Authors:** Fatemeh Shahmoradi Ghaheh, Milad Razbin, Majid Tehrani, Leila Zolfipour Aghdam Vayghan, Mehdi Sadrjahani

**Affiliations:** 1 Department of Textile Engineering, Faculty of Environmental Science, Urmia University of Technology, Urmia, Iran; 2 School of Engineering, Macquarie University, Sydney, NSW 2109 Australia; 3 Department of Textile Engineering, Amirkabir University of Technology, Tehran, Iran; 4 Department of Art, Shahrekord University, Shahrekord, 5681188617 Iran

**Keywords:** Dyeing process, Plum-tree leaves, Artificial neural network, Response surface methodology, Genetic algorithm, Synthesis and processing, Computational science

## Abstract

The dyeing process of textile materials is inherently intricate, influenced by a myriad of factors, including dye concentration, dyeing time, pH level, temperature, type of dye, fiber composition, mechanical agitation, salt concentration, mordants, fixatives, water quality, dyeing method, and pre-treatment processes. The intricacy of achieving optimal settings during dyeing poses a significant challenge. In response, this study introduces a novel algorithmic approach that integrates response surface methodology (RSM), artificial neural network (ANN), and genetic algorithm (GA) techniques for the precise fine-tuning of concentration, time, pH, and temperature. The primary focus is on quantifying color strength, represented as K/S, as the response variable in the dyeing process of polyamide 6 and woolen fabric, utilizing plum-tree leaves as a sustainable dye source. Results indicate that ANN (R^2^ ~ 1) performs much better than RSM (R^2^ > 0.92). The optimization results, employing ANN-GA integration, indicate that a concentration of 100 wt.%, time of 86.06 min, pH level of 8.28, and a temperature of 100 °C yield a K/S value of 10.21 for polyamide 6 fabric. Similarly, a concentration of 55.85 wt.%, time of 120 min, pH level of 5, and temperature of 100 °C yield a K/S value of 7.65 for woolen fabric. This proposed methodology not only paves the way for sustainable textile dyeing but also facilitates the optimization of diverse dyeing processes for textile materials.

## Introduction

In recent years, with increasing concern about environmental issues, the use of natural dyes for textile dyeing has been increased. Natural dyes are biodegradable and prevent the accumulation of toxic waste in the environment^1^. Beyond their compatibility with the environment, certain natural dyes also possess additional benefits, such as anti-allergic and anti-bacterial properties^2,3^. However, despite good properties of such natural dyes, not many companies have used them commercially. The problem with using natural dyes is, their poor color fastness, low color strength and limited shades. To solve the mentioned problems, new natural dyes, new bio-based mordants and changing the effective parameters in the dyeing process such as dyeing method, concentration of dye, type and concentration of mordant, dyeing time as well as temperature and pH of the dyeing process, can be used^3,4^.

In recent researches, natural mordants are introduced as an alternative to metal mordants. Some natural materials, such as the pomegranate rind, red sumac, pinecone and peppermint due to the presence of phenolic groups in their structure, have performed very well as bio-mordant in the dyeing process of textiles^2,3,5,6^. Using bio-based mordant reduces the destructive effects of metal mordants on the environment. Bio-based mordants are also of medical importance as they possess anti-bacterial and anti- allergic properties^3,7^. Investigating the effect of dyeing parameters on textiles dyed with natural dyes reveals that optimal parameter values are crucial for color quality. Recent research using modeling methods has identified optimal dyeing parameters. Kuo et al. combined Artificial Neural Network (ANN) and Genetic Algorithm (GA) to predict color strength (K/S) of polyester and Lycra^®^ fabric dyed with Everacid Red RFL^8^. Rosa et al. used a central composite design with 26 experiments, focusing on temperature, NaCl, Na_2_CO_3_, NaOH, processing time, and RB5 concentration to maximize K/S in cotton fabric. They combined ANN and PSO to improve the dyeing process, demonstrating cost-reduction potential for the industry^9^. Ghanmi et al. assessed the predictive capabilities of Fuzzy Logic and Response Surface Methodology (RSM) for dyeing wool and polyamide fibers with Juglans R. extract. They considered extract concentration, dyeing time, and temperature, with K/S as the response variable. Fuzzy Logic showed the lowest error values, outperforming RSM in predicting dyeing behavior^10^. Pervez et al*.* used a Taguchi L27 design to predict multiple responses, including exhaustion percentage (E%), fixation rate (F%), total fixation efficiency (T%), and K/S of cotton fabric dyed with Reactive Blue 194. They controlled dye concentration, fixing temperature, fixing time, pH, material-to-liquor ratio, and salt concentration. support vector machine (SVR) showed superior predictive capabilities with an R^2^ value of 0.9819^11^. Haji and Vadood used GA, particle swarm optimization (PSO), and gray wolf optimization (GWO) to optimize models predicting color coordinates (L*, a*, and b*) of cotton fabrics dyed with aluminum potassium sulfate and natural dyes (weld and madder). They found that ANN combined with GWO provided the highest accuracy for predicting L* and b*, while ANN with PSO was best for predicting a*^12^. Abdelileh et al. used ANN to predict K/S and dye bath exhaustion of acrylic fiber dyed with Indigo Carmine. They employed RSM, with dye concentration, pH, temperature, and time as control factors. ANN showed high accuracy in predictions, while RSM excelled in optimizing the dyeing process^13^.

Despite the advantages mentioned for natural dyes, the supply of these dyes is only 1% of world demand. This shows that there is immense scope to venture into search for other sources of natural dyes. Plums are the most taxonomically diverse of stone fruits tree and are adapted to a board range of climatic and edaphic factors. They are placed within the *Pronoideae* subfamily of the Rosaceae, in the subgenus Prunophora and includes several species of Prunus. The most commonly grown species are P. domestica L., Prunus Americana and P. salicina Lindl^14^. The plum tree has leaves with different colors. In one type of Prunus Americana, the leaves are brownish-red. In terms of material novelty, for the first time a sustainable procedure using plum-tree leaves to dyeing polyamide 6 and woolen fabrics is proposed in this work. Moreover, we employed RSM to formulate an experimental design incorporating control factors such as concentration, time, pH, and temperature. The primary objective was to quantify K/S as the response variable in the dyeing process of polyamide 6 and woolen fabric utilizing plum-tree leaves as the dye source. Subsequently, we used two distinct methodologies, namely ANN and RSM, to elucidate the relationship between the aforementioned control factors and the response variable. To enhance the optimization process, a GA was seamlessly integrated into both objective functions, aiming to refine the control factors and maximize the response variable. The resultant optimized set of control factors was then applied in the dyeing process, facilitating a comparative analysis between optimized and non-optimized samples. To deepen the analysis, the impact of bio-mordants on color intensity was examined.

## Experimental section

### Materials

In this study, two common used fabrics made up of polyamide 6 and woolen fibers have been purchased from the Iranian Company with specification as summarized in Table 1. Before starting the dyeing or mordanting procedure, the fabrics were immersed in an aqueous solution of non-ionic detergent (2 mL/L of Lissapol NC) for 30 min at a temperature of 70 °C to remove dirt. The scoured fabrics were then thoroughly washed with tap water, followed by drying at room temperature.

**Table 1:** Fabrics specification.

Fiber	Pattern	Pattern density (thread/cm)		Yarn count (N_m_)		Areal density
Polyamide 6	Plain	18	14	25	36	60
Woolen	Plain	26	31	47	56	180

Collection of plum-tree leaves from local gardens (Khoy-Urmia city) was done; following washing and drying, they were turned in to the powder form through milling. Five biomordants, including *Carthamus Tinctorius*, *Terminalia Chebula*, *Rhus Coriaria L*., *Urtica*, and *Juglans R* were bought from an herbal pharmacology store in Iran, then grinded to obtain a fine powder. Metal mordants such as copper sulfate, zinc sulfate, potassium dichromate and iron III sulfate were purchased from Merck, Germany. Acetic acid (Merck, Germany) and sodium hydroxide (Kohan Taj Kimia, Iran) were used to adjust pH in the dyeing process.

### Mordanting and dyeing procedure

Pre-mordanting method was used for dyeing procedure. For mordanting, the sample was entered into the mordant solution; after that, it was brought to heating. The fabrics were mordanted at 100 °C for 75 min using 5 wt.% metal mordants and 50 wt.% biomordants solution as well as the liquor ratio being 50:1. The aqueous extract were utilized for dyeing both polyamide 6 and woolen fabrics.

### Design of experiment and dyeing procedure

For the parametric study and data collection, we have chosen four variables including concentration, time, pH, and temperature. To ensure accuracy, we have specified the lower and upper limits for each design parameter. Table 2 provides the unit, code, and these limits for the design parameters.

**Table 2:** Design parameters of experiment.

Factor	Unit	Code	Lower limit	Upper limit
Concentration	wt.%	X_1_	20	100
Time	min	X_2_	30	120
pH	–	X_3_	5	9
Temperature	$^\circ C$	X_4_	40	100

The Box Behenken method, a subgroup of RSM with a center point equal to 5, was utilized under Design Expert Software to generate various combinations of design parameters. This method assumes three levels for each design parameter and effectively reduces the total number of combinations from 81 to 25. Table 3 provides a summary of the design matrices used in the experiments.

**Table 3:** Design matrices of the experiment.

Run	Control parameters				Response		Dataset of fold 1	
	X_1_	X_2_	X_3_	X_4_	Y_1_	Y_2_	Training	Testing
1	20	75	7	40	1.19 $\pm$ 0.01	1.31 $\pm$ 0.01	+	−
2	20	75	5	70	3.85 $\pm$ 0.04	1.47 $\pm$ 0.01	+	−
3	20	30	7	70	2.11 $\pm$ 0.02	1.28 $\pm$ 0.01	+	−
4	20	120	7	70	3.36 $\pm$ 0.02	2.2 $\pm$ 0.02	+	−
5	20	75	9	70	2.33 $\pm$ 0.01	1.72 $\pm$ 0.01	+	−
6	20	75	7	100	2.24 $\pm$ 0.03	1.86 $\pm$ 0.2	+	−
7	60	75	5	40	3.07 $\pm$ 0.05	1.65 $\pm$ 0.01	+	−
8	60	30	7	40	3.11 $\pm$ 0.03	1.88 $\pm$ 0.01	+	−
9	60	120	7	40	2.56 $\pm$ 0.02	2.15 $\pm$ 0.01	+	−
10	60	75	9	40	2.4 $\pm$ 0.03	1.81 $\pm$ 0.01	−	+
11	60	30	5	70	4.38 $\pm$ 0.07	1.8 $\pm$ 0.01	+	−
12	60	120	5	70	7.19 $\pm$ 0.09	5.59 $\pm$ 0.05	−	+
13	60	75	7	70	5.59 $\pm$ 0.04	2.51 $\pm$ 0.02	+	−
	60	75	7	70				
	60	75	7	70				
	60	75	7	70				
	60	75	7	70				
14	60	30	9	70	2.78 $\pm$ 0.03	2.52 $\pm$ 0.02	+	−
15	60	120	9	70	5.11 $\pm$ 0.06	3.44 $\pm$ 0.03	+	−
16	60	75	5	100	7.46 $\pm$ 0.08	3.19 $\pm$ 0.03	+	−
17	60	30	7	100	6.01 $\pm$ 0.05	2.79 $\pm$ 0.02	+	−
18	60	120	7	100	7.82 $\pm$ 0.11	5.04 $\pm$ 0.04	+	−
19	60	75	9	100	6.84 $\pm$ 0.07	5.38 $\pm$ 0.05	+	−
20	100	75	7	40	4.28 $\pm$ 0.06	2.55 $\pm$ 0.02	−	+
21	100	75	5	70	7.82 $\pm$ 0.08	2.55 $\pm$ 0.02	+	−
22	100	30	7	70	5.04 $\pm$ 0.06	2.79 $\pm$ 0.03	+	−
23	100	120	7	70	9.04 $\pm$ 0.16	3.12 $\pm$ 0.03	+	−
24	100	75	9	70	7.69 $\pm$ 0.05	4.31 $\pm$ 0.06	+	−
25	100	75	7	100	9.23 $\pm$ 0.19	5.27 $\pm$ 0.05	+	−

### Characterization methodologies

The reflectance spectra of dyed samples were acquired through the utilization of a Konica Minolta 3600d within the visible light range spanning from 400 to 700 nm with increments of 10 nm. Subsequently, the K/S for the samples was determined by employing the Kubelka–Munk equation, as delineated in Eq. (1).

1 $$\frac{K}{S}=\frac{{\left(1-R\right)}^{2}}{2R}$$

Herein, $R$ represents the reflectance value at each wavelength, with $K$ denoting the absorbance coefficient and $S$ representing the scattering coefficient. Furthermore, the CIEL*a*b coordinates of the samples were computed under CIE standard illuminant D65 and 10˚ standard observer in CIE 1964. Determination of washing color fastness was done in accordance with ISO 105-C06 (A1S): 2010 test method. Evaluation of the change in color of the specimen was done using gray scale (rating from 1 to 5) and ISO 105-A02. The standard ISO 105-B02:2013 and a blue scale (rating from 1 to 8) were used for the purpose of evaluating the dyed fabrics' light fastness. Color fastness to dry rubbing and wet rubbing fastness were tested in accordance with ISO 105-X12: (2016) test method using a manually operated Crockmeter and grey scale.

## Soft computation

### Data pre-processing and post-processing

In the process of constructing a model based on specific data, evaluating the correlation between variables is imperative to circumvent multicollinearity. Multicollinearity arises when two or more independent variables in a model exhibit substantial correlation. This heightened correlation introduces challenges to the model, such as unreliable coefficients, diminished statistical significance, augmented standard errors, escalated variance inflation factor (VIF), and increased model instability^15^. Consequently, the utilization of the Pearson’s correlation coefficient (PCC) through Eq. (2) was deemed essential for this purpose.

2 $$PCC=\frac{\sum ({X}_{i}-\overline{X })({Y}_{i}-\overline{Y })}{\sqrt{\sum {({X}_{i}-\overline{X })}^{2}\sum {({Y}_{i}-\overline{Y })}^{2}}}$$

**Figure 1:**
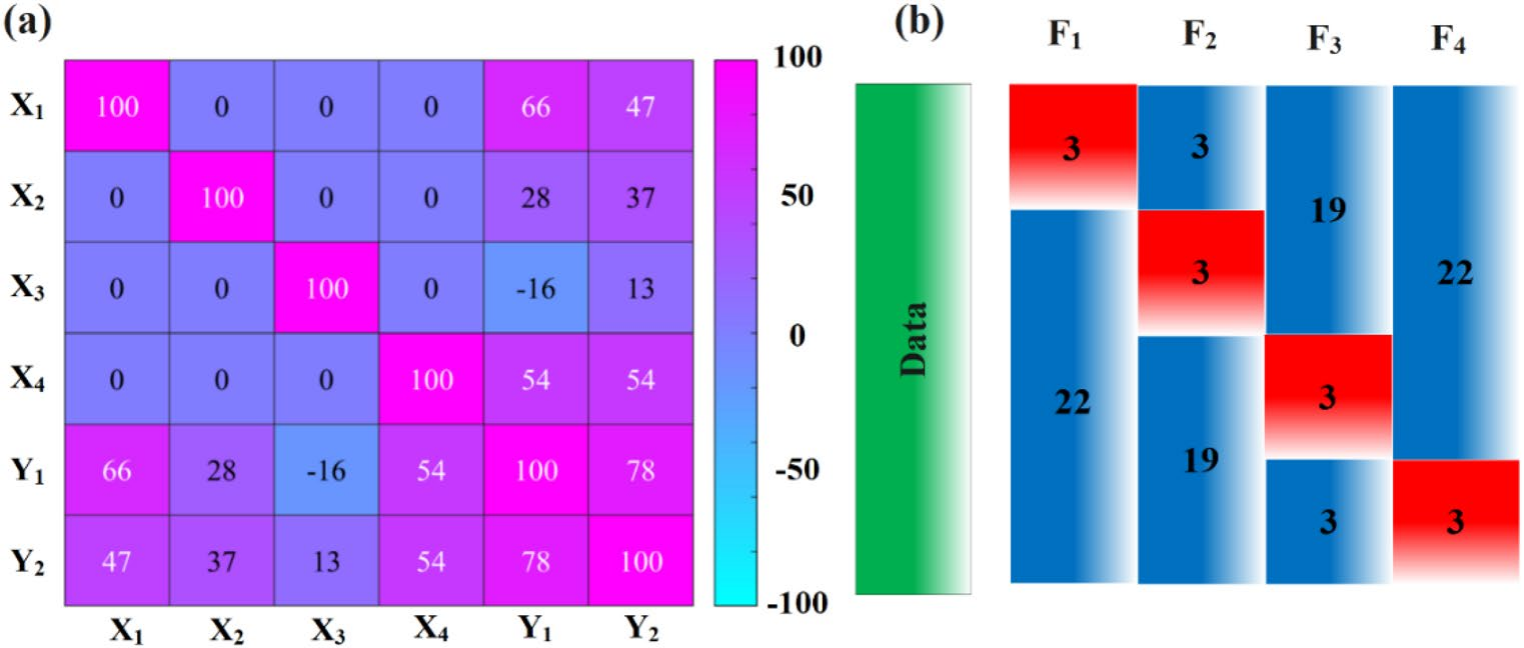
(**a**) Pearson’s heatmap correlation matrix of data, and (**b**) fourfold cross-validation of data with a data split ratio of 9:1.

By calculating Eq. (2) for the gathered data, Fig. 1a, is obtained. It is evident from the results that the correlations among the input data are notably low. Conversely, the correlations among the output data exhibit an acceptable level (78%). In general, if the PCC value surpasses 90%, it is advisable to exclude one of the correlated parameters during the modeling and optimization stages to mitigate redundancy. However, in the present study, all parameters were retained during modeling and optimization steps due to their PCC values falling below the critical threshold.

It is imperative to normalize experimental data prior to formulating the objective function to enhance the efficacy of the model. When data exhibit variations across different scales, quantitative interactions among them can be compromised. Therefore, normalization proves advantageous in mitigating scale sensitivity, expediting convergence, facilitating gradient descent, preventing issues related to vanishing or exploding gradients, enhancing generalization, and ensuring numerical stability^16^. Equation (3) is employed for the normalization of all data, encompassing both control factors and response variables, as an essential preprocessing step before the modeling phase.

3 $${X}_{n}=(a-b)\frac{X-{X}_{min}}{{X}_{max}-{X}_{min}}+b$$

where $a$ and $b$ denote the upper (= 0.9) and lower (= 0.1) limits of the normalization domain, respectively. Subsequent to the normalization process, a division of the data was executed with a split ratio of 90:10, creating two distinct datasets comprising training and testing groups. Following this, a fourfold cross-validation method was employed to assess both the generality and average performance of the objective function as shown in Fig. 1b.

### Modeling step

To optimize the hyper parameters of objective functions, encompassing ANN and RSM, a criterion is essential to evaluate model performance during the training phase. Previous research^17^ has demonstrated that the efficacy of models developed through either ANN or RSM can be quantified using a metric known as the Total Goodness Function (TGF). The TGF comprehensively assesses the performance of the objective function across both training and testing phases. Moreover, it amalgamates the coefficient of determination and mean squared error, enhancing its robustness. Mathematically, the determination of TGF is articulated as follows:

4 $$TGF=\frac{{n}_{train}}{N}{({R}^{2}+{e}^{-MSE})}_{train}+\frac{{n}_{test}}{N}{({R}^{2}+{e}^{-MSE})}_{test}$$

In which

5 $${R}^{2}=1-\frac{\sum_{1}^{n}{(s-p)}^{2}}{\sum_{1}^{n}{(s-\overline{s })}^{2}}$$

6 $$MSE=\frac{\sum_{1}^{n}{(s-p)}^{2}}{n}$$

In the aforementioned expression, the symbols represent the following: $e$ designates the actual value, $p$ denotes the predicted value, $\overline{e }$ signifies the average of the actual values, $n$ stands for the number of data points, and $N$ represents the total number of data points.

#### Response surface methodology

RSM proves to be a highly efficacious approach not only in the design of experiments but also as a robust method for formulating objective functions and conducting optimization procedures. It furnishes a range of polynomial equations that serve to establish a meaningful relationship between control factors and the response variable. Broadly, RSM is advantageous for streamlined optimization, comprehensive exploration of factor interactions, minimization of required experimental runs, precise quantification of optimal conditions, rigorous statistical analysis, graphical representation of response surfaces, robustness testing, systematic sequential optimization, accommodation of nonlinear relationships, and judicious resource utilization^18^. In the present study, four distinct control factors are under consideration, each influencing a singular response variable. Mathematically, the expression of the RSM model is articulated as follows:

7 $$\widehat{f}\left({x}_{1}.\dots .{x}_{4}\right)={a}_{0}+\sum_{1}^{4}{a}_{i}{x}_{i}+\sum_{1}^{4}{a}_{ii}{{x}_{i}}^{2}+\sum_{1}^{4}\sum_{1}^{4}{a}_{ij}{x}_{i}{x}_{j}$$

where ${a}_{0}$, ${a}_{i}$, ${a}_{ii}$, and ${a}_{ij}$ are offset, linear coefficient, quadratic coefficient and linear–linear interaction coefficient of model, respectively. It is noteworthy to highlight that, owing to the employment of diverse criteria for assessing the performance of the RSM model, the Solver toolbox within the Excel software was employed to derive the offset and coefficient values.

#### Artificial neural network

**Figure 2:**
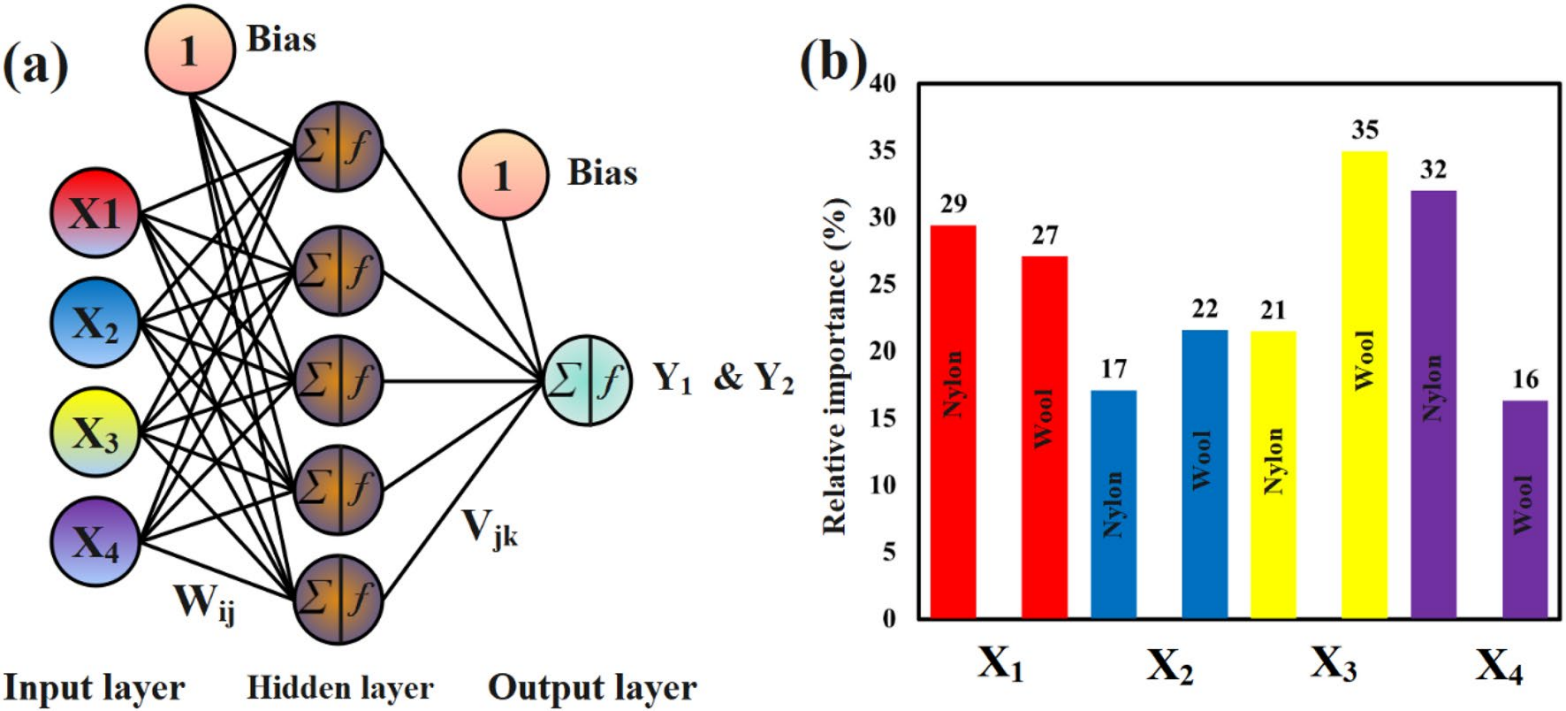
(**a**) Topology of developed artificial neural network, (**b**) relative importance of input parameter in networks.

In the domain of modeling, the ANN, particularly the feed-forward back-propagation architecture, stands out as a potent network for establishing relationships between control factors and response variables. Diverging from RSM, an ANN model can be structured to map from multiple factors to multiple response variables. It is noteworthy that both ANN and RSM lack differentiability. Consequently, vector-based optimization methods, such as genetic algorithms (GAs), particle swarm optimization (PSO), simulated annealing, differential evolution (DE), ant colony optimization (ACO), Bayesian optimization, grid search and random search, hybrid methodologies, and covariance matrix adaptation evolution strategy (CMA-ES), have been explored to address this challenge^19,20^. In the present study, the optimal model was identified with a neural network topology of 4–5-1 as shown in Fig. 2a, demonstrating the highest Total Goodness Function (TGF) value, quantified at 2. The training process employed a learning rate of 0.6 and a momentum value of 0.9, determined as optimized parameters. The Levenberg–Marquardt algorithm was utilized to train the neural network efficiently. Additionally, the transfer functions for the hidden layer and output layer were designated as Eq. (8) and (9), respectively.

8 $$tansig\left(y\right)=\frac{2}{\left(1+{e}^{-2y}\right)}-1$$

9 $$purelin\left(y\right)=y$$

In this context, the variable $y$ represents the sum of weights multiplied by signals from various nodes, along with the inclusion of a bias value. It is imperative to emphasize that the fine-tuning of hyperparameters for the ANN model was conducted through a dedicated program implemented in MATLAB, employing the grid search method. Likewise, the Total Goodness Function (TGF) served as the criterion for discerning the most optimal performing network. To elucidate the relative significance of control parameters, a sensitivity analysis was employed as outlined below^21^:

10 $${R}_{i}(\%)=\frac{\sum_{j=1}^{{n}_{H}}\left[\frac{\left|{W}_{ij}\right|\left|{V}_{j}\right|}{\sum_{l=1}^{{n}_{I}}\left|{W}_{lj}\right|}\right]}{\sum_{i=1}^{{n}_{I}}\left[\sum_{j=1}^{{n}_{H}}\left[\frac{\left|{W}_{ij}\right|\left|{V}_{j}\right|}{\sum_{l=1}^{{n}_{I}}\left|{W}_{lj}\right|}\right]\right]}\times 100$$

The weight between the i-th node in the input layer and the j-th node in the hidden layer is denoted as ${W}_{ij}$, and the weight between the j-th node in the hidden layer and the output layer is denoted as ${V}_{j}$​. Here, ${n}_{I}$​ represents the number of nodes in the input layer, and ${n}_{H}$​ represents the number of nodes in the hidden layer. Figure 2b illustrates the outcomes of the sensitivity analysis. In the case of the network developed for polyamide 6 fabric, the control parameter X_4_ has the highest contribution at 32%, while the control parameter X_2_ has the lowest contribution at 17%. On the other hand, in the woolen fabric network, the control parameters X_3_ and X_4_ have contributions of 35% and 16% respectively, impacting the output.

### Development of cost function

In engineering, the establishment of objective functions enables optimization processes, which involve determining a set of parameters for purposes such as maximization, minimization, and objective optimization. Optimization is a fundamental element in engineering design, analysis, and decision-making^22,23^. This research focuses on maximizing color strength, denoted as K/S, representing the ratio of light absorbed (K) to scattered (S) by a material. In the context of textiles, enhancing K/S is advantageous for multiple reasons. A higher K/S value signifies increased light absorption, contributing to a more profound and vibrant color appearance, particularly significant in the textile industry where fabric aesthetics strongly influence consumer preferences. Elevated K/S also enhances resistance to fading, allowing manufacturers to create textiles with prolonged color intensity despite exposure to environmental factors. Fabrics with heightened K/S are associated with superior quality, leading to increased consumer demand. Consistency in maintaining a high K/S is vital for uniformity in textile appearance, meeting quality standards, and fulfilling customer expectations. For textile manufacturers and fashion brands, achieving vibrant and enduring colors contributes to a positive brand image, fostering consumer loyalty in competitive industries where superior K/S provides a distinct advantage. Consumers actively seek products with enduring aesthetic appeal, making fabrics with superior K/S highly desirable. Utilizing a GA as an optimization tool necessitates defining the cost function, expressed as the negative normalized value of color strength of polyamide 6 fabric, as detailed in Eqs. (11).

11 $$Minimize:f\left(\overrightarrow{x}\right)=-{{Y}_{nylon}}_{n}(\overrightarrow{x})$$

For woolen fabric, we have

12 $$Minimize:g\left(\overrightarrow{x}\right)=-{{Y}_{woolen}}_{n}(\overrightarrow{x})$$

13 $$\overrightarrow{x}=\left\{{{x}_{1}}_{i},{{x}_{2}}_{i},{{x}_{3}}_{i},{{x}_{4}}_{i}\right\} i=1.\dots . k$$

where $\overrightarrow{x}$ is a vector to store control parameters, with their upper and lower limits aligning with the boundaries specified in the control parameters table.

### Optimization

The GAs are computational optimization and search technique inspired by the process of natural selection and genetics. It is a heuristic algorithm used to find approximate solutions to optimization and search problems. Developed based on the principles of evolution, genetic algorithms are particularly effective for solving complex problems where traditional optimization methods may struggle. The basic idea behind a GA is to mimic the process of natural selection by evolving a population of potential solutions to a problem over multiple generations. The algorithm starts with a population of individuals, each representing a potential solution, and applies genetic operators such as selection, crossover, and mutation to produce new generations of individuals. Over successive generations, the algorithm tends to converge towards solutions that are better suited to the optimization problem. GAs are versatile and have been applied to a wide range of optimization problems, including engineering design, scheduling, financial modeling, and machine learning. Their effectiveness lies in their ability to explore a large search space efficiently and find near-optimal solutions in complex and non-linear problem domains^24^. In the context of this research, a population size of 100 individuals was established, with a termination criterion defined as 50 generations. The generation of individuals involved the application of a uniform function, while the scaling of these individuals was executed using a fitness scaling function. Parental selection was achieved through a roulette function, incorporating a two-point crossover function with a fraction of 0.8, and introducing a viable mutation with a fraction of 0.2. Furthermore, migration was permitted bidirectional with a fraction of 0.7.

**Figure 3:**
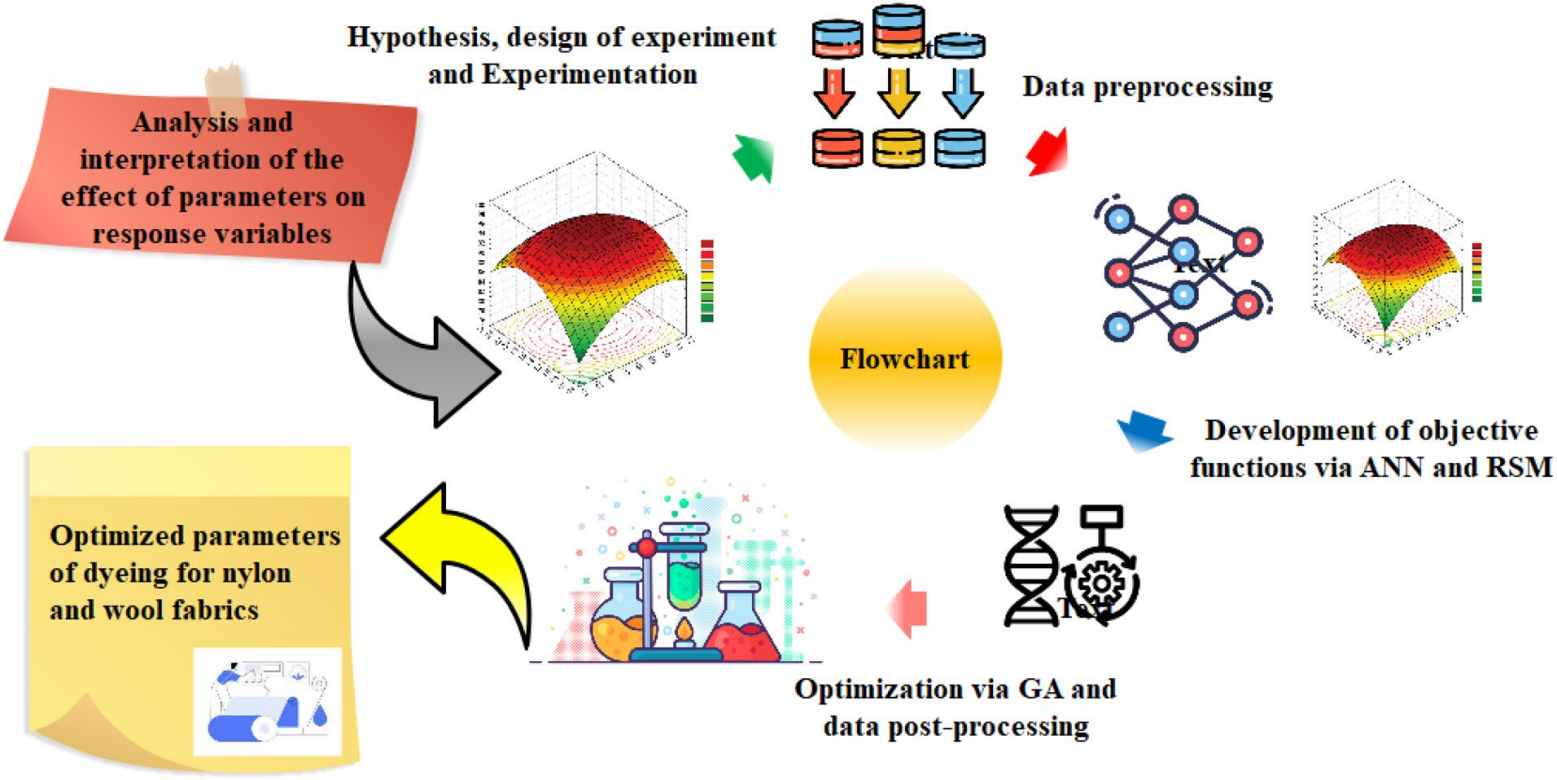
Flowchart of developed multi-approach procedure.

Figure 3 illustrates the flowchart of the devised multi-approach procedure. The process commences with an analysis and interpretation of the impact of control parameters on the response variables. Subsequently, in accordance with the formulated hypotheses, an experiment is designed and executed. Following the normalization and shuffling of data, two methodologies, namely ANN and RSM, are employed to construct the objective functions. Thereafter, GA is integrated with both models, and optimization procedures are conducted. Lastly, the optimized dyeing parameters are utilized for dyeing polyamide 6 and woolen fabrics with plum-tree leaves.

## Results and discussions

### Statistical analysis

The significance of various terms in the RSM models are determined through Analysis of Variance (ANOVA). The results of the ANOVA analysis are summarized in Table 4, reflecting a 95% confidence level for the developed RSM models. Notably, several terms in both RSM models exhibit p-values higher than 0.05, suggesting that they could be excluded. Nevertheless, these terms were retained to enhance the performance of the RSM models.

**Table 4:** ANOVA analysis of different terms of RSM-based models.

Source	D. F	S. S		M. S		P-value		Coefficient-value	
		Polyamide 6	Wool	Polyamide 6	Wool	Polyamide 6	Wool	Polyamide 6	Wool
Model	14	135.59	36.97	9.68	2.64	0.00	0.00	0.1156	0.2060
${x}_{1}$	1	65.43	9.63	65.43	9.63	0.00	0.00	0.2080	0.2116
${x}_{2}$	1	11.31	5.99	11.31	5.99	0.00	0.00	0.1089	0.1661
${x}_{3}$	1	3.65	0.72	3.65	0.72	0.07	0.22	− 0.1778	− 0.4176
${x}_{4}$	1	44.05	12.36	44.05	12.36	0.00	0.00	0.3873	− 0.7974
${{x}_{1}}^{2}$	1	1.00	0.48	1.00	0.48	0.33	0.32	− 0.2420	− 0.3158
${{x}_{2}}^{2}$	1	0.80	0.40	0.80	0.40	0.38	0.36	− 0.2144	0.2293
${{x}_{3}}^{2}$	1	0.02	0.81	0.02	0.81	0.90	0.20	− 0.0276	0.3539
${{x}_{4}}^{2}$	1	2.65	0.52	2.65	0.52	0.12	0.29	− 0.3958	0.3337
${x}_{1}{x}_{2}$	1	1.89	0.09	1.89	0.09	0.19	0.66	0.4275	− 0.1713
${x}_{1}{x}_{3}$	1	0.48	0.57	0.48	0.57	0.49	0.27	0.2163	0.4380
${x}_{1}{x}_{4}$	1	3.80	1.18	3.80	1.18	0.07	0.12	0.5902	0.7427
${x}_{2}{x}_{3}$	1	0.06	2.06	0.06	2.06	0.81	0.05	− 0.0951	− 0.6001
${x}_{2}{x}_{4}$	1	1.39	0.98	1.39	0.98	0.25	0.16	0.3669	0.5742
${x}_{3}{x}_{4}$	1	0.00	1.03	0.00	1.03	0.98	0.15	0.0093	0.5804
Residual	14	13.62	6.18	0.97	0.44				
Total	10	149.20	43.15						

D.F is the degree of freedom, S.S is the adjusted sum of squares, and M.S is the adjusted means squares.

### Selection of objective function

**Figure 4:**
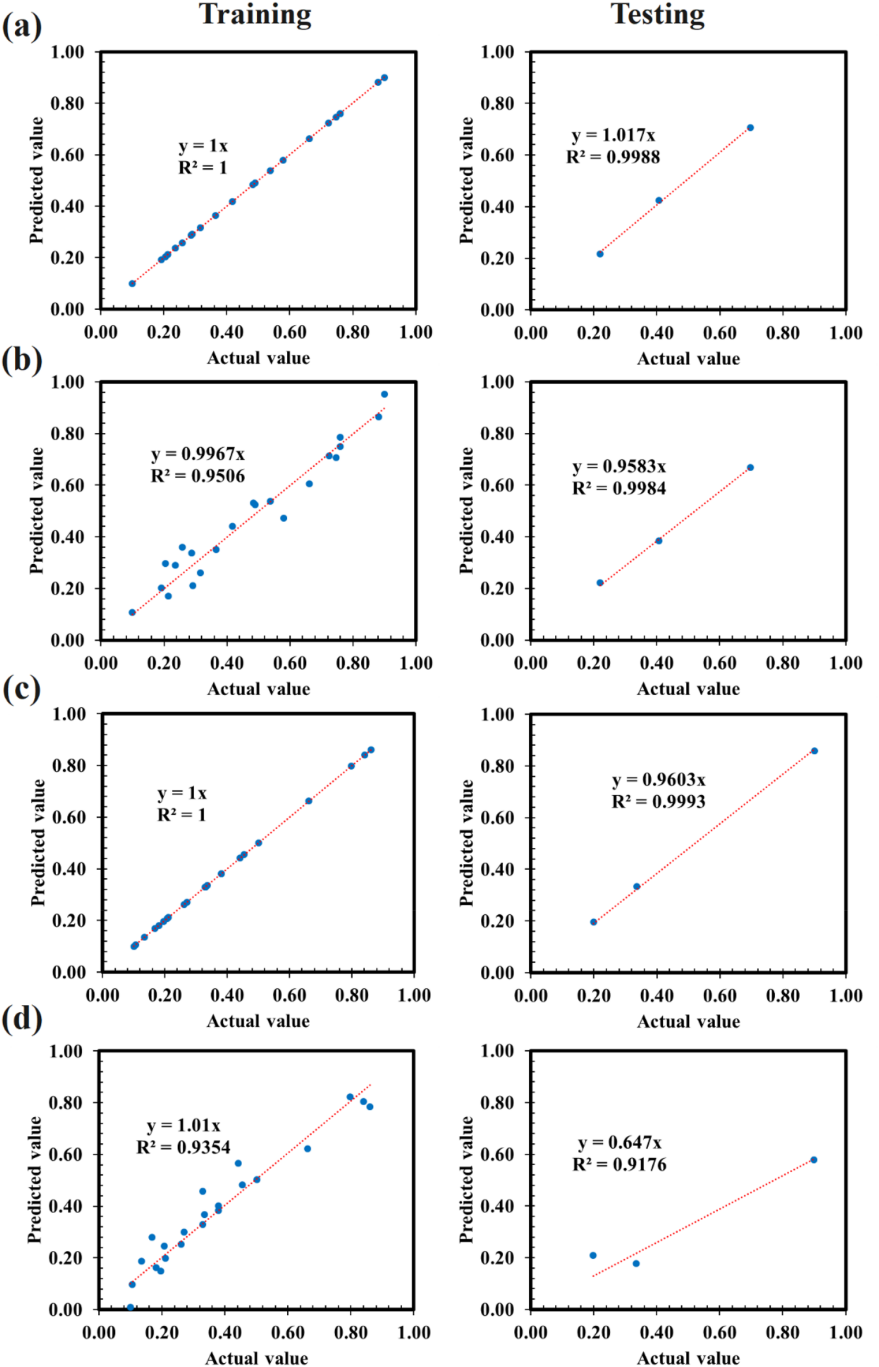
Pearson correlation heat map matrix of (**a**) training and testing step of ANN, (**b**) training and testing step of RSM model for polyamide 6 fabric (**c**) training and testing step of ANN, and (**d**) training and testing step of ANN for woolen fabric.

The optimization step benefits from a procedure yielding the best-performing objective function. Remarkably, both ANN models yielded nearly identical models with a goodness of fit of 1, as depicted in Fig. 4a–c while RSM models suffered from lack of fit due to complexity of the relationship between parameters as shown in Fig. 4b–d. A R^2^ value of 1 signifies a perfect prediction of the response variable based on control parameters, explaining 100% of the variability. While this scenario is rare and may suggest overfitting, caution is warranted with extremely high R^2^ values, as they may indicate poor generalization to new data. In practice, R^2^ values typically range between 0 and 1, signifying the model's fit to the data. However, high R^2^ alone does not ensure predictive ability on new data; additional metrics and techniques, such as cross-validation, are essential for assessing generalization performance. A fourfold cross-validation demonstrated consistent model performance across all folds.

The weight and bias values of the developed ANN models of polyamide 6 and woolen fabrics are presented in Tables 5 and 6, respectively. Employing these identical values within a network characterized by fixed weight and bias parameters facilitates the replication of the outcomes depicted in Figure.

**Table 5:** Weight and bias values of the ANN model for polyamide 6 fabric.

Weight						Bias	
W	− 1.9081	− 0.3914	0.6955	− 0.7066		1	− 0.1550
	− 1.6402	− 0.6561	− 2.1239	− 0.0170			1.0360
	1.0881	0.4659	− 0.6768	1.3734			0.7681
	− 0.6385	− 1.9450	1.1608	− 3.2947			− 0.9855
	− 0.3262	1.1239	1.2294	− 1.5772			− 1.5330
V	− 0.8986	− 0.2427	− 1.0823	− 0.7352	− 0.3494	2	− 0.0304

**Table 6:** Weight and bias values of the ANN model for woolen fabric.

Weight						Bias	
W	1.5727	1.0439	1.8911	2.7729		1	− 3.2672
	0.0312	− 2.1175	− 0.1403	0.6810			− 0.2401
	− 4.0678	− 0.6270	0.5521	− 1.9067			− 1.5018
	− 0.8151	− 0.3422	− 1.0256	− 0.0315			− 1.9647
	− 1.2034	1.8901	− 2.3527	0.9119			− 4.0079
V	0.6950	0.0751	− 0.2046	− 1.0171	1.2517	2	0.3994

### Optimization results

**Figure 5:**
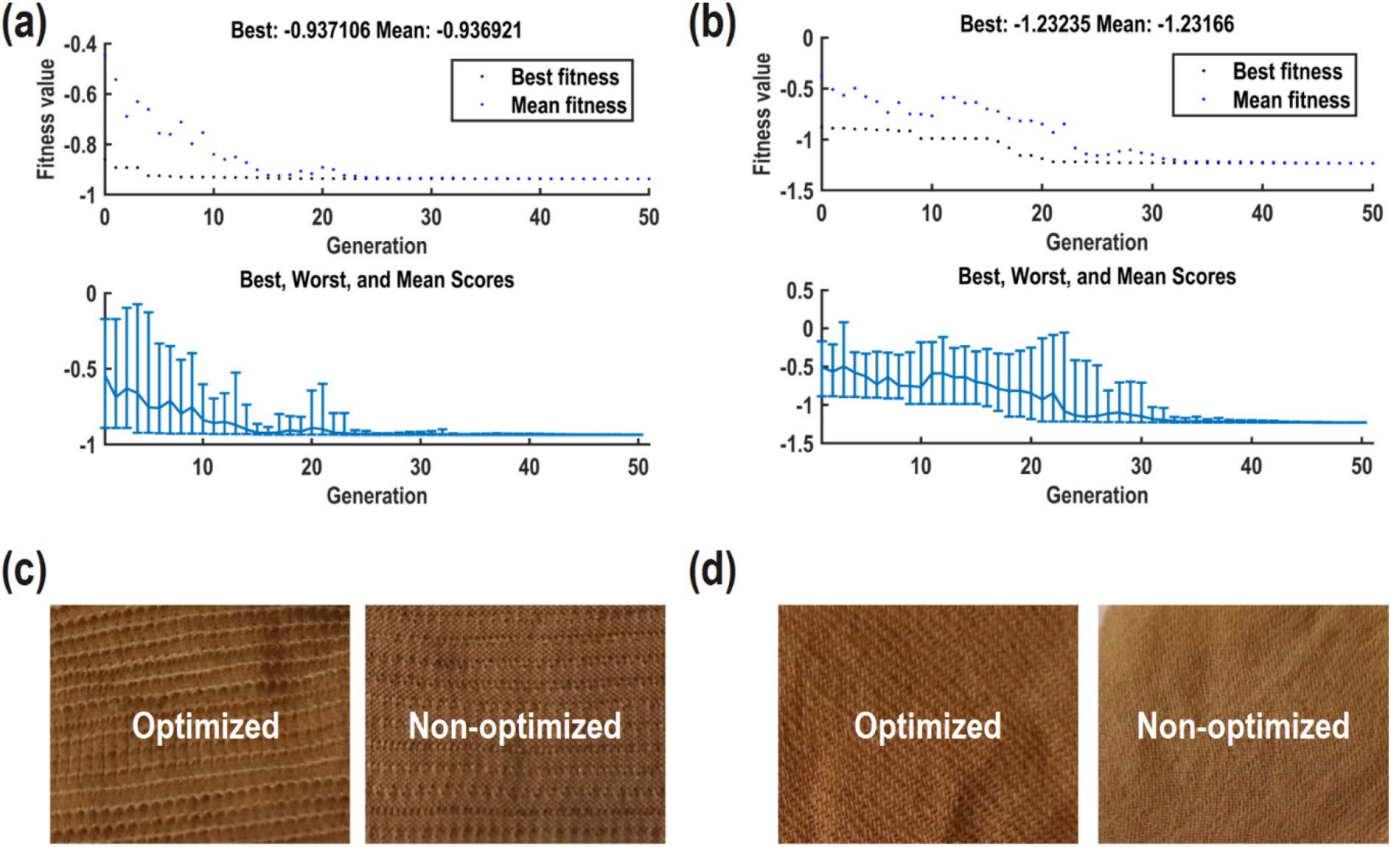
Optimization performance of (**a**) ANN-GA model of polyamide 6 fabric, (**b**) ANN-GA model of woolen fabric, (**c**) visual comparison between optimized and non-optimized polyamide 6 fabric, (**d**) visual comparison between optimized and non-optimized woolen fabric.

Figure 5a,b depict the optimization performance of ANN-GA of polyamide 6 and woolen fabrics, respectively. Both models exhibit significant diversity during the optimization process and converge after 50 generations. The convergence of GAs implies that a state has been reached where further iterations do not yield substantial improvements in performance or optimization objectives. This stabilization is associated with the algorithms finding solutions meeting specified criteria. Figure 5c,d compares the visual appearance of optimized and non-optimized samples, revealing that the optimized sample has a higher K/S value.

In Table 7, a comparison between GA and DOE-guided samples in predicted and experimental scenarios has been made. In the case of polyamide 6 fabric, the GA-guide sample experienced an increase in time from 75 to 86 min, and an increase in pH from 7 to 8.28. The concentration (100 wt.%) and temperature (100 °C) remained consistent. The error of blind prediction of K/S with an experimental value of 10.21 is acceptable, with a value of 5.97%. For the woolen fabric, the concentration declined from 60 wt.% to 55.85 wt.%, while the temperature increased from 70 °C to 99.79 °C. The maximum K/S was 7.65 with a prediction error of 3.52%.

**Table 7:** Comparison between GA and DOE-guided samples in predicted and experimental scenarios.

Sample	Method	Input parameter				K/S		Error (%)
		X_1_	X_2_	X_3_	X_4_	Predicted	Experimental	
Polyamide 6	GA-Guided	100.00	86.06	8.28	100.00	9.60	10.21	5.97
	DOE-Guided	100.00	75.00	7.00	100.00	9.23	9.23	0.00
Wool	GA-Guided	55.85	120.00	5.00	99.97	7.38	7.65	3.52
	DOE-Guided	60.00	120.00	5.00	70.00	5.59	5.59	0.00

### Interpretation of results

#### The effect of design parameter on the color strength

**Figure 6:**
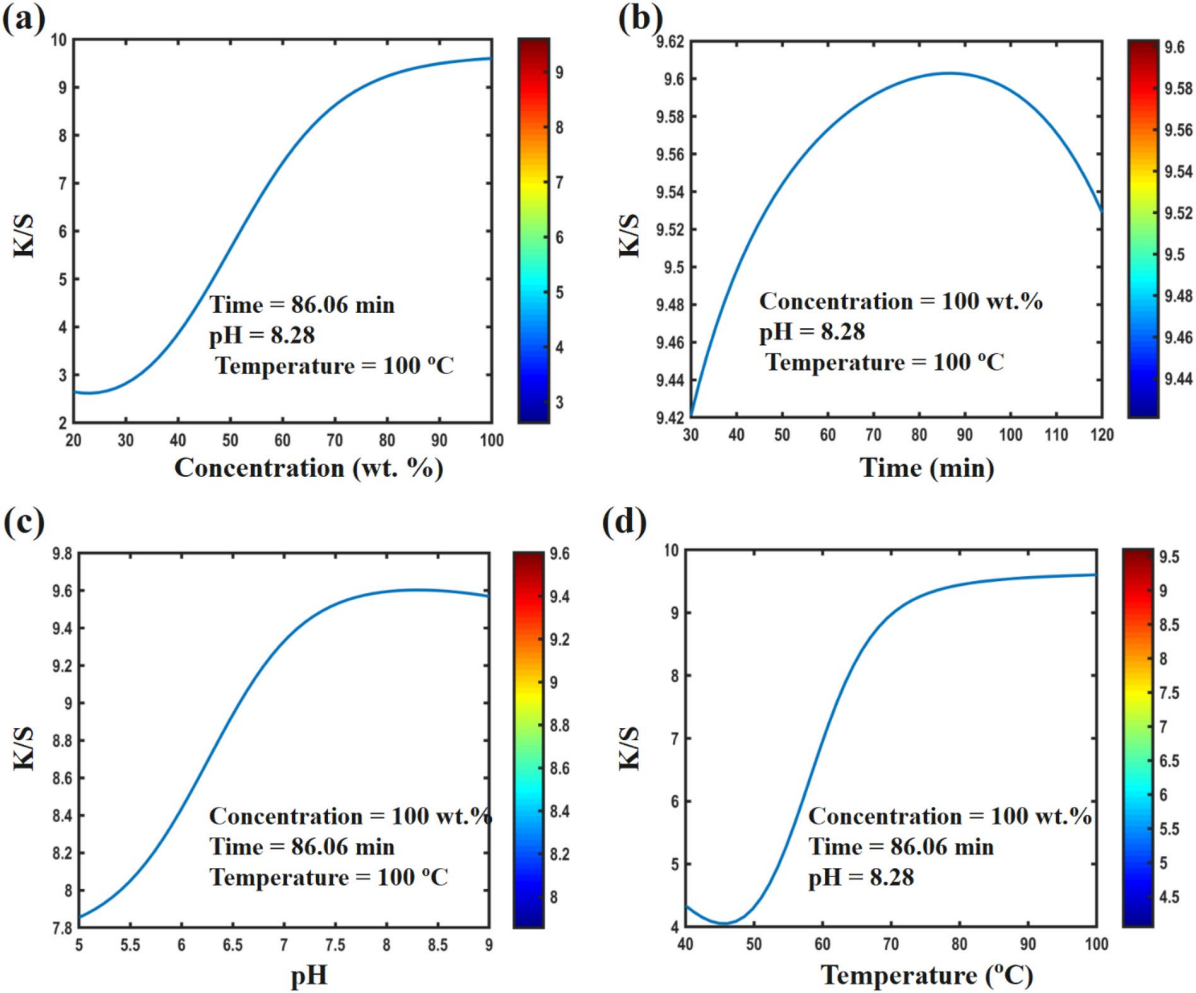
Effect of design parameters on K/S value of polyamide 6 fabric calculated by ANN for optimized sample, (**a**) concentration, (**b**) time, (**c**) pH, and (**d**) temperature.

**Figure 7:**
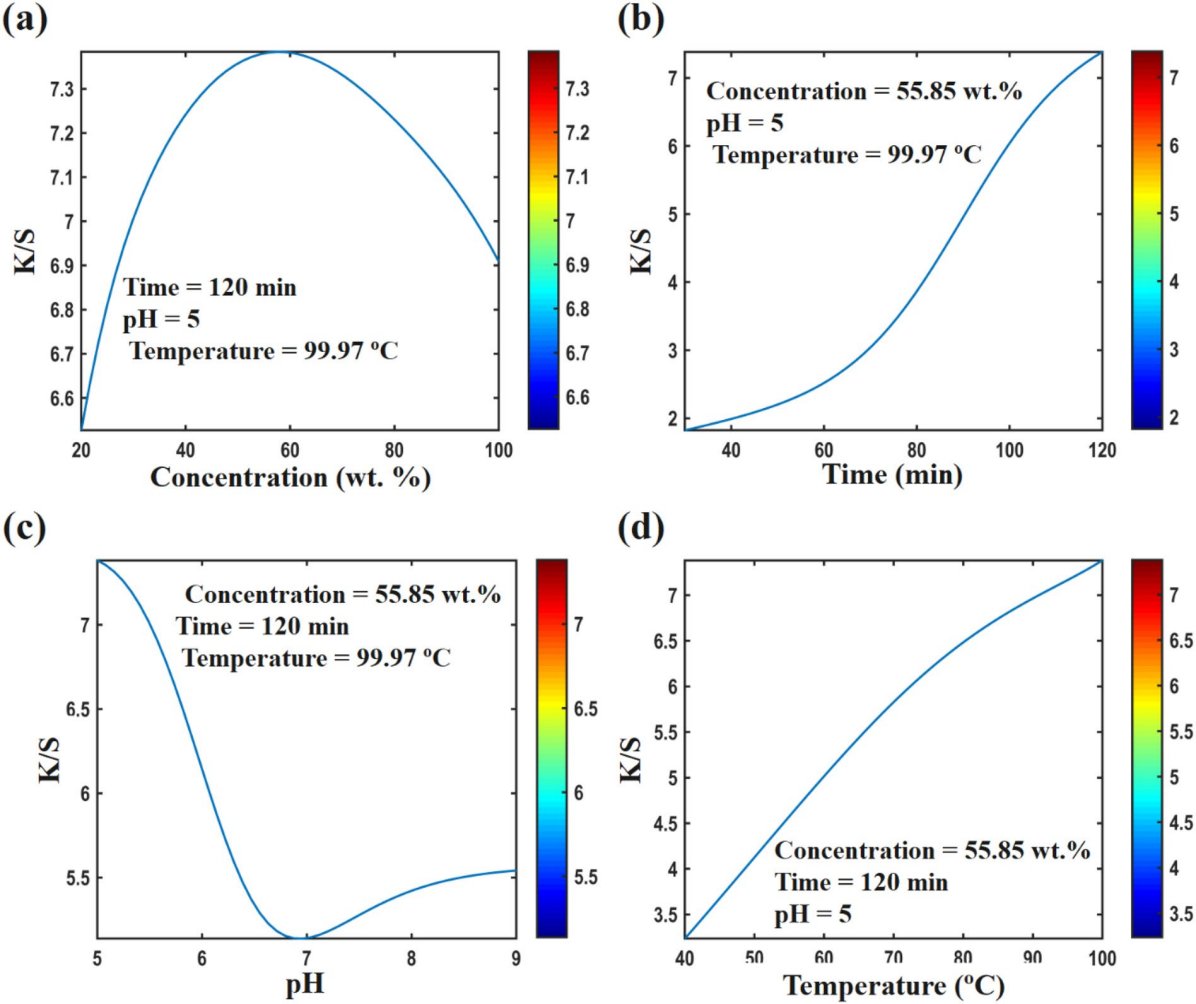
Effect of design parameters on K/S value of woolen fabric calculated by ANN for optimized sample, (**a**) concentration, (**b**) time, (**c**) pH, and (**d**) temperature.

Investigating the effect of dyeing parameters on the color strength of polyamide 6 and woolen dyed with plum-tree leaves show that in order to create color with good quality, each effective parameter in dyeing process have to be set in a certain value. Figure 6a shows that increasing the dye concentration up to 90 wt.% has significantly increased the color strength of the dyed polyamide 6 fabrics. An increasing the dye concentration more than 90 wt.%, had very little effect on the color strength. Also, Fig. 7a shows that increasing the dye concentration up to 55.85 wt.% has increased the color strength of dyed wool fabrics. Increasing the dye concentration more than 55.85 wt.% has reduced the absorption and color strength in woolen fibers. Using more than the proposed value of the dye concentration might lead to over-exhaustion, causing adsorption of dye on the fiber’s surface and poor fixation of color on the polyamide 6 fibers. Figure 6b shows that increasing the dyeing time up to 86.06 min has caused a slight increase in the color strength on polyamide 6 fibers. Increasing the dyeing time more than 86.06 min has not had a significant effect on the color strength. The results of the effect of dyeing time on wool fibers are different. Figure 7b shows that by increasing the dyeing time up to 120 min, the color strength has increased, significantly. In Figs. 6c and 7c, the effect of pH on the color strength is given. Figure 6c shows that the maximum dye absorption in polyamide 6 fibers was around the neutral region (pH level of 8.28). In wool fibers, with a decrease in pH level, the color strength has increased, significantly. Figures 6d and 7d show that increasing the temperature in the dyeing process up to 80 °C in polyamide 6 fibers and up to 100 °C in wool fibers has increased the color strength.

In general, the above results show that the best dyeing conditions to create the highest color strength in polyamide 6 fibers are 100 wt.% dye concentration, 86.06 min dyeing time, pH level of 8.28 and 100 °C dyeing temperature. Also, the highest color strength in wool fibers is created at 55.85 wt.% dye concentration, 120 min dyeing time, pH level of 5 and 100 °C dyeing temperature.

**Figure 8:**
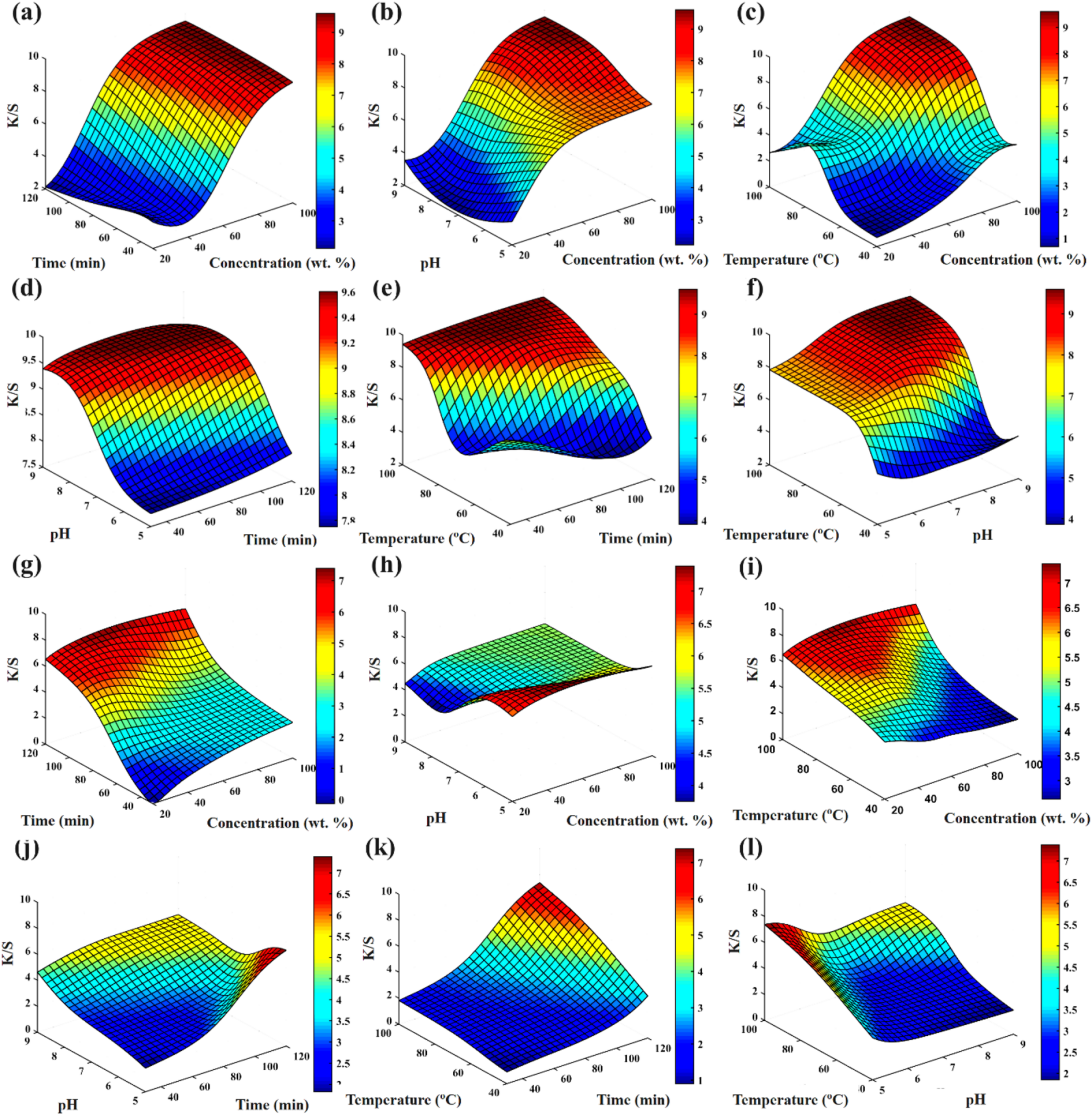
3D plot of interaction between design parameters of K/S value for polyamide 6 fabric, (**a**) concentration vs time, (**b**) concentration vs pH, (**c**) concentration vs temperature, (**d**) time vs pH, (**e**) time vs temperature, (**f**) pH vs temperature, and K/S value for woolen fabric, (**g**) concentration vs time, (**h**) concentration vs pH, (**i**) concentration vs temperature, (**j**) time vs pH, (**k**) time vs temperature, and (**l**) pH vs temperature.

In Fig. 8, the mutual influence of dye concentration, dyeing time, pH level and dyeing temperature on the dye absorption in polyamide 6 and wool fibers are shown. The results indicate that all the investigated dyeing parameters were effective on the dye absorption. Among the examined parameters, dyeing temperature and dye concentration were the most effective parameters on the dye absorption in polyamide 6 fibers, respectively. In polyamide 6 fibers, the dyeing time had the least effect on the absorption. Increasing the temperature of the dyeing bath causes swelling in the polyamide 6 fibers, and as a result, the ligands in the polyamide 6 fiber are easily accessible to dye molecules. Also, increasing the temperature has reduced the dye accumulation and increased the dye permeation and dye absorption in polyamide 6 fibers^25^.

pH level and dye concentration have the highest effect on the dye absorption in wool fibers. In wool fibers, the temperature had the least effect on the dye absorption. The use of acids and the reduction of pH in the dyeing process causes the hydrolysis of the Ligand group in the plum tree leaves. In this case, the affinity of these groups to connect with metal or natural mordant (especially with coordinate covalent), increases. In addition, the acidic condition facilitates the effective transfer of electrons from the carboxyl and amino groups in the wool structure to the used mordants and the formation of coordinate covalent bonds between these group^26^.

#### The effect of bio-mordants on the color strength

**Figure 9:**
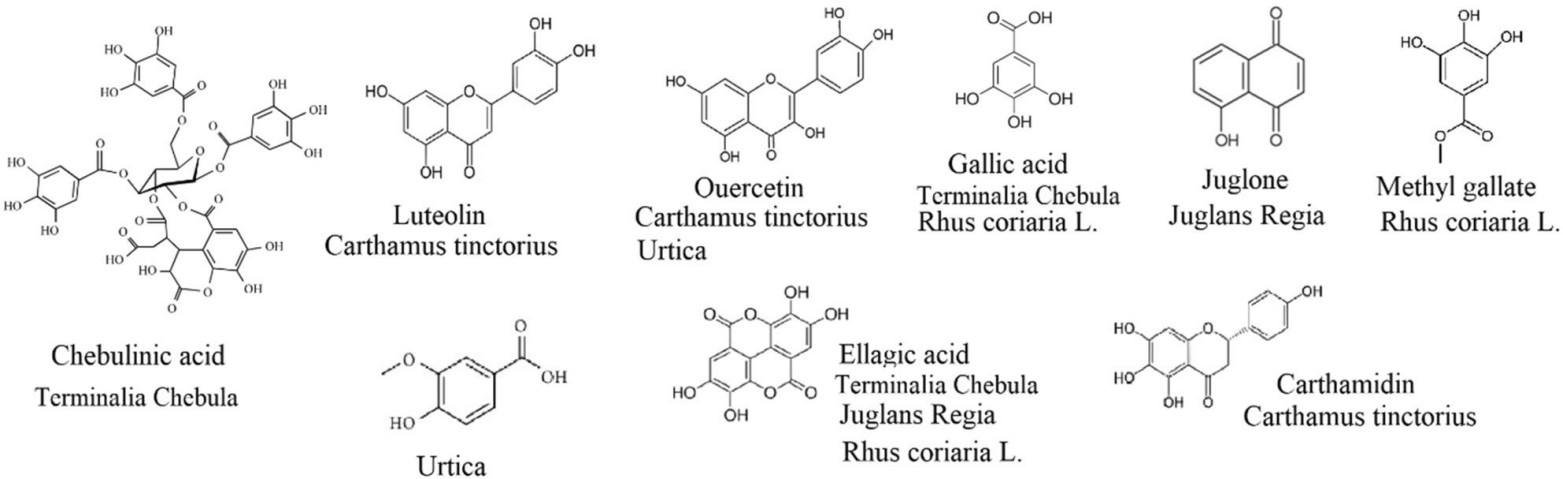
The major constituents of used biomordants^29–32^.

In dyeing with natural dyes, in order to achieve an appropriate colorfast shades, it is necessary to treat the fibers with mordants. Traditionally, metal salts such as Al^3+^, Cu^2+^, Cr^2+^, and Fe^2+^ have been used as mordant. Some of these metals such as Cu and Cr are toxic and dangerous and have harmful effects on aquatic life, water quality and soil fertility^27^. Therefore, there is a growing trend to replace these harmful metals with eco-friendly and sustainable materials such as plant phenolic materials^3,5,6^. In this study, metal salts like Fe, Cu, Cr, and Zn as well as such phenolic extract from *Juglans R*, *Rhus coriaria L*., *Terminalia chebula*, *Carthamus tinctorius*, and *Urtica* were used to act as mordant. Due to the presence of hydroxyl and carboxyl groups in the structure of these plant materials (See Fig. 9), the biomolecules interact with the ligands in the polyamide 6 and wool fibers as well as the dye, forming additional H-bonds that help create new colorfast shades^28^. In addition, the composition of these biomolecules enhances the development of darker shades with stable hues, making them an attractive alternative to toxic metal mordants.

In Tables 8, 9, 10, 11, present the colorimetric and fastness results for dyed polyamide 6 and woolen fabrics, both without mordants and with metal and plant mordants. The a* and b* values indicate that different mordants affect these parameters, but there was no shift in the a*-b* quadrant of the CIELab color space. Both control and mordanted samples fall within the red-yellow quadrant of the CIELab color space.

**Table 8:** Shade quality parameters of polyamide 6 fabric dyed with metal and bio-mordants.

Mordant used	Dyed polyamide 6 fabric	L*	a*	b*	C*	h°	ΔE	K/S (λ_max_)
Without Mordant		52.65	4.82	19.00	19.60	75.75	–	8.04
Cr		54.62	3.48	33.25	33.43	84.03	14.45	8.00
Zn		55.13	3.57	20.38	20.69	80.05	3.11	7.42
Cu		55.18	4.25	23.19	23.57	79.61	4.93	7.56
Fe		54.51	2.77	20.33	20.52	82.25	3.08	7.71
Juglans Regia		34.43	7.19	15.08	16.70	64.51	18.78	13.26
Rhus coriaria L		47.57	6.38	15.21	16.49	67.26	6.52	7.79
Terminalia Chebula		49.53	4.74	21.30	21.83	77.46	3.88	10.01
Carthamus tinctorius		48.94	3.97	26.94	27.23	81.61	8.81	10.52
Urtica		53.30	2.97	19.49	19.72	81.35	2.03	7.71

**Table 9:** Fastness grading of polyamide 6 fabric dyed with metal and bio-mordants.

Mordant used	Light fastness	Wash fastness	Staining wash fastness-viscose	Staining wash fastness- wool	Dry rubbing fastness	Wet rubbing fastness
Without Mordant	3–4	3	3–4	3–4	3–4	3
Cr	6–7	5	4–5	5	4–5	4
Zn	4–5	3–4	4	3–4	4	4
Cu	6	4–5	5	4–5	5	4–5
Fe	6–7	4	4–5	4	5	5
*Juglans R*	8	5	5	5	5	5
*Rhus coriaria L*	6	4–5	4–5	5	4–5	4
*Terminalia chebula*	7–8	5	5	5	5	5
*Carthamus tinctorius*	6–7	4–5	5	4–5	4–5	4
*Urtica*	6–7	4–5	5	5	4–5	4

**Table 10:** Shade quality parameters of wool fabric dyed with metal and biomordants.

Mordant used	Dyed wool fabric	L*	a*	b*	C*	h°	ΔE	K/S (λ_max_)
Without Mordant		50.95	10.31	19.16	27.76	61.71	–	7.71
Cr		37.03	7.88	33.57	34.48	76.78	20.18	16.38
Zn		52.21	9.36	20.68	22.7	65.65	2.19	7.13
Cu		41.21	8.57	22.95	24.5	69.53	10.6	11.74
Fe		40.71	3.98	13.77	14.33	73.88	13.19	9.71
*Juglans R*		37.47	9.47	15.99	18.59	59.36	13.88	11.91
*Rhus coriaria L*		44.78	7.93	17.11	18.86	65.15	6.93	9.68
*Terminalia Chebula*		48.37	8.26	28.04	29.23	73.59	9.47	13.99
*Carthamus tinctorius*		48.52	10.29	30.08	31.79	71.12	11.19	11.43
*Urtica*		42.76	12.28	19.2	22.79	57.4	8.43	10.65

**Table 11:** Fastness grading of wool fabric dyed with metal and bio-mordants.

Mordant used	Light fastness	Wash fastness	Staining wash fastness-viscose	Staining wash fastness-wool	Dry rubbing fastness	Wet rubbing fastness
Without Mordant	3	2–3	4	3–4	4	3
Cr	5	5	5	5	4–5	4–5
Zn	4	3	3–4	3–4	4	4
Cu	5	3–4	4	3–4	5	4–5
Fe	7	4–5	4–5	4	5	5
*Juglans R*	8	5	5	5	5	5
*Rhus coriaria L*	7–8	5	5	5	4–5	4
*Terminalia Chebula*	7	5	5	5	5	5
*Carthamus tinctorius*	6	5	5	5	4	4
*Urtica*	7	4–5	5	5	4–5	4–5

In polyamide 6 fibers, the Juglans Regia sample exhibited significantly higher darkness and redness compared to other samples. For these fibers, Cr had the highest yellowness, while Juglans Regia and Rhus coriaria L. had the least. In wool fibers, Cr and Juglans Regia samples showed greater darkness than other metal and bio-mordant samples. Carthamus tinctorius and Urtica samples displayed the highest redness, whereas Cr, Carthamus tinctorius, and Terminalia Chebula samples had the highest yellowness.

Examining the color difference (ΔE) reveals that among metal mordants, Cr, and among bio-mordants, Juglans Regia, exhibited the most significant color change compared to the non-mordanted sample.

Additionally, the non-mordanted sample demonstrated adequate color strength (8.04 in polyamide 6 and 7.71 in wool) when compared to the mordanted samples. However, its washing, rubbing, and light fastness were significantly lower than those of the mordanted samples. According to ISO standards, the washing, rubbing, and light fastness of the non-mordanted sample are unacceptable (wash and rubbing fastness ≤ 4 and light fastness ≤ 5).

In polyamide 6 fibers, the color strength of the mordaned samples with *Juglans R* (13.26), *Terminalia chebula* (10.01) and *Carthamus tinctorius* (10.52) was higher than the mordanted samples with metal salts (7.42–8.00). Also, the wash, rubbing, and light fastness of these samples was higher than or equal to the fastness of the mordanted samples with metals. In polyamide 6 fibers, the mordanted samples with *Rhus coriaria* L. and *Urtica* had the same color strength and fastness as the metal ones. In woolen fibers, the sample mordanted with Cr had the highest color strength (16.38). The wash and rubbing fastness of the Cr sample is equal to the bio-mordant samples, but the light fastness is much lower. The used bio-mordants contain polyphenol components (see Fig. 9). Phenolic structures absorb UV and improve the light fastness^33^. In woolen fibers, after the Cr sample, the mordanted sample with *Terminalia chebula* had the highest color strength (13.99). The color strength of the other biomordanted samples was in the range of metal ones (except Cr sample). Also, the wash, rubbing, and light fastness of biomordanted samples was equal to or more than metal ones. The wash fastness of all biomordanted samples were the same as sample (5). However, the light fastness of *Juglans R* (8) and *Rhus coriaria* L. (7–8) samples was higher than the other ones. Also, the rubbing fastness of *Juglans R* (5) and *Terminalia Chebula* (5) samples was higher than or equal to the metal samples.

In this research, although the use of bio-mordants has produced hues with appropriate color strength and color fastness, in some cases, such as with Juglans Regia, these mordants have altered the original hue obtained from the colorant (see ΔE in Tables 8 and 10). To address this issue, it is suggested to use bio-mordants without coloring properties.

## Conclusion

This study focused on conducting an experimental inquiry into the influence of concentration, time, pH, and temperature on the K/S of polyamide 6 and woolen fabrics utilizing plum-tree leaves. The experiment was structured through response surface methodology. To establish a mapping function from control parameters to the response variable, both ANN and RSM were employed. Following the development of the objective function, a GA was integrated into both objective functions. Subsequently, optimization procedures were carried out, and the optimized set of control parameters were applied to dye polyamide 6 fabric using plum-tree leaves. The findings can be summarized as follows:

- RSM not only facilitates the exploration of the parametric influence of control factors but also establishes a data space characterized by significant diversity.
- ANN exhibits superior generalization to the data space of the dyeing process compared to response surface methodology.
- The integration of genetic algorithms (GA) with ANN allows for fine-tuning the dyeing process of polyamide 6 and woolen fabrics with plum-tree leaves, contributing to enhanced optimization.
- By optimizing the dyeing conditions and using plant materials as bio-mordant, it is possible to obtain hues with excellent color strength as well as washing and light fastness on polyamide 6 and wool fabrics. This approach provides a sustainable and eco-friendly method of dyeing polyamide 6 and woolen fabrics, which replaces the need for synthetic dyes and toxic metal mordants. Textile industries can adopt greener practices by using plant dyes and mordants while ensuring the durability and vibrancy of the dyed fabrics.

## Data Availability

The data that support the findings of this study are available on request from the corresponding authors.

## References

[1] Desai, J., Chauhan, J., Mankad, A. & Maitreya, B. Natural colourants: A review. *Int. Assoc. Biol. Computat. Digest* **1**(2), 261–270 (2023).

[2] Yameen, M. *et al.* Sustainable eco-friendly extraction of yellow natural dye from haar singhar (nyctanthes arbor-tritis) for bio coloration of cotton fabric. *Environ. Sci. Pollut. Res.* **55**(29), 83810–83823 (2022). 10.1007/s11356-022-21450-0 35771330

[3] Shahmoradi Ghaheh, F., Moghaddam, M. K. & Tehrani, M. Comparison of the effect of metal mordants and bio-mordants on the colorimetric and antibacterial properties of natural dyes on cotton fabric. *Color. Technol.* **6**(137), 689–698 (2021).

[4] Rehman, F. U. *et al.* Microwave-assisted exploration of yellow natural dyes for nylon fabric. *Sustainability* **9**(14), 5599 (2022).

[5] Tehrani, M., Ghaheh, F. S., Beni, Z. T. & Rahimi, M. Extracted dyes’ stability as obtained from spent coffee grounds on silk fabrics using eco-friendly mordants. *Environ. Sci. Pollut. Res.* **26**(30), 68625–68635 (2023). 10.1007/s11356-023-27157-0 37126177

[6] Haji, A., Shahmoradi Ghaheh, F. & Mohammadi, L. Dyeing of polyamide 6 fabric with new bio-colorant and bio-mordants. *Environ. Sci. Pollut. Res.* **13**(30), 37981–37996 (2023). 10.1007/s11356-022-24862-0 36575254

[7] Naveed, R. *et al.* Microwave assisted extraction and dyeing of cotton fabric with mixed natural dye from pomegranate rind (punica granatum l.) and turmeric rhizome (curcuma longa l.). *J. Nat. Fibers* **1**(19), 248–255 (2022).

[8] Kuo, C.-F., Chang, C.-D., Su, T.-L. & Fu, C.-T. Optimization of the dyeing process and prediction of quality characteristics on elastic fiber blending fabrics. *Polym.-Plast. Technol. Eng.* **7**(47), 678–687 (2008).

[9] Rosa, J. M. *et al.* Modeling and optimization of reactive cotton dyeing using response surface methodology combined with artificial neural network and particle swarm techniques. *Clean Technol. Environ. Policy* **23**, 2357–2367 (2021).

[10] Ghanmi, H., Sebeia, N., Jabli, M., Al-Ghamdi, Y. O. & Algohary, A. M. Insight into fuzzy logic and response surface methodologies for predicting wool and polyamide dyeing behaviors with a biological extract of juglans regia. *Fibers Polym.* **12**(23), 3473–3481 (2022).

[11] Pervez, M. N. *et al.* Optimization and prediction of the cotton fabric dyeing process using taguchi design-integrated machine learning approach. *Scie. Rep.* **1**(13), 12363 (2023). 10.1038/s41598-023-39528-1 PMC10390507 37524835

[12] Haji, A. & Vadood, M. Prediction of color coordinates of cotton fabric dyed with binary mixtures of madder and weld natural dyes using artificial intelligence. *Fibers Polym.* **5**(24), 1759–1769 (2023).

[13] Abdelileh, M. *et al.* Dyeing of modified acrylic fibers with indigo carmine: Modeling and optimization of the dyeing process using a combination of rsm and ann methodologies. *Fibers Polym.* **7**(24), 2377–2389 (2023).

[14] Ertekin, C., Gozlekci, S., Kabas, O., Sonmez, S. & Akinci, I. Some physical, pomological and nutritional properties of two plum (prunus domestica l.) cultivars. *J. Food Eng.* **4**(75), 508–514 (2006).

[15] Marwala, T. & Leke, C. A. *Handbook of Machine Learning: Volume 2: Optimization and Decision Making* (World Scientific, 2019).

[16] Dutka, A. F. & Hansen, H. H. *Fundamentals of Data Normalization* (Addison-Wesley Longman Publishing Co., Inc., 1991).

[17] Haghdoost, F., Razbin, M., Bahrami, H., Barzin, J. & Ghaee, A. Modeling and optimization of the core-shell nanofibrous composite mat as a scaffold via hybrid models. *J. Ind. Text.* **52**, 15280837221112406 (2022).

[18] Davim, J. P. & Aveiro, P. *Design of Experiments in Production Engineering* (Springer, 2016).

[19] Fausett, L. V. *Fundamentals of Neural Networks: Architectures, Algorithms and Applications* (Pearson Education India, 2006).

[20] Sadeghi, M. R., Hosseini Varkiyani, S. M. & Asgharian Jeddi, A. A. Machine learning in optimization of nonwoven fabric bending rigidity in spunlace production line. *Sci. Rep.* **1**(13), 17702 (2023). 10.1038/s41598-023-44571-z PMC10582177 37848503

[21] Sohrabi, M., Razbin, M., Pourtavvaf, M., Bagherzadeh, R. & Mehdipour Mirmahale, M. Exercising hybrid model to design an optimized electrospun polyamide-6 nanofibrous mat for air filtration applications. *J. Text. Inst.* **11**(114), 1667–1681 (2023).

[22] Amor, N., Noman, M. T., Petru, M., Mahmood, A. & Ismail, A. Neural network-crow search model for the prediction of functional properties of nano tio2 coated cotton composites. *Sci. Rep.* **1**(11), 13649 (2021). 10.1038/s41598-021-93108-9 PMC8249465 34211049

[23] Amor, N., Noman, M. T., Petru, M. & Sebastian, N. Comfort evaluation of zno coated fabrics by artificial neural network assisted with golden eagle optimizer model. *Sci. Rep.* **1**(12), 6350 (2022). 10.1038/s41598-022-10406-6 PMC9012820 35428810

[24] Hatami, O., Sayadi, D., Razbin, M. & Adibi, H. Optimization of grinding parameters of tool steel by the soft computing technique. *Computat. Intell. Neurosci.* **2022**, 3042131 (2022). 10.1155/2022/3042131 PMC9763009 36544858

[25] Amin, N. *et al.* Sustainable application of cochineal-based anthraquinone dye for the coloration of bio-mordanted silk fabric. *Environ. Sci. Pollut. Res.* **27**, 6851–6860 (2020). 10.1007/s11356-019-06868-3 31879870

[26] Herath, D., Wickramasinghe, G., Aponsu, G. & Perera, V. Effects of acidification of clove fruit dye extracted in water and ethanol for performance enhancement of dsscs. *Sri Lankan J. Phys.*10.4038/sljp.v22i1.8097 (2021).

[27] Manian, A. P. The role of mordants in fixation of natural dyes. In *Handbook of Natural Colorants* (eds Stevens, C. *et al.*) 507–515 (Wiley, 2023).

[28] Kupnik, K., Primožič, M., Vasić, K., Knez, Ž & Leitgeb, M. A comprehensive study of the antibacterial activity of bioactive juice and extracts from pomegranate (punica granatum l.) peels and seeds. *Plants* **8**(10), 1554 (2021). 10.3390/plants10081554 PMC8402121 34451599

[29] Jahanban-Esfahlan, A., Ostadrahimi, A., Tabibiazar, M. & Amarowicz, R. A comprehensive review on the chemical constituents and functional uses of walnut (juglans spp.) husk. *Int. J. Mol. Sci.* **16**(20), 3920 (2019). 10.3390/ijms20163920 PMC6719079 31409014

[30] Batiha, G.E.-S. *et al.* Rhus coriaria l.(sumac), a versatile and resourceful food spice with cornucopia of polyphenols. *Molecules* **16**(27), 5179 (2022). 10.3390/molecules27165179 PMC9414570 36014419

[31] Upadhyay, A., Agrahari, P. & Singh, D. A review on the pharmacological aspects of terminalia chebula. *Int. J. Pharmacol* **6**(10), 289–298 (2014).

[32] Asgarpanah, J. & Kazemivash, N. Phytochemistry, pharmacology and medicinal properties of carthamus tinctorius l. *Chin. J. Integr. Med.* **19**, 153–159 (2013). 23371463 10.1007/s11655-013-1354-5

[33] Souissi, M., Guesmi, A. & Moussa, A. Valorization of natural dye extracted from date palm pits (phoenix dactylifera) for dyeing of cotton fabric. Part 2: Optimization of dyeing process and improvement of colorfastness with biological mordants. *J. Clean. Prod.* **204**, 1143–1153 (2018).

